# Effect of an Online Mobility Self-Management Program on Walking Speed in Older Adults With Preclinical Mobility Limitation: Protocol for a Randomized Controlled Trial

**DOI:** 10.2196/72585

**Published:** 2025-05-21

**Authors:** Julie Richardson, Ayse Kuspinar, Susanne Sinclair, Marla Beauchamp, Sinead Dufour, Ada Tang, Joy MacDermid, Evelyne Durocher, Lehana Thabane, Feng Xie, Andrew Costa

**Affiliations:** 1 School of Rehabilitation Science Faculty of Health Sciences McMaster University Hamilton, ON Canada; 2 McMaster Institute for Research on Aging (MIRA) McMaster University Hamilton, ON Canada; 3 Department of Health Research Methods, Evidence, and Impact Faculty of Health Sciences McMaster University Hamilton, ON Canada; 4 Michael G. DeGroote School of Medicine Faculty of Health Sciences McMaster University Hamilton Canada; 5 McMaster Physical Activity Centre of Excellence (PACE) Faculty of Science McMaster University Hamilton Canada; 6 School of Physical Therapy Faculty of Health Sciences Western University London, ON Canada; 7 Canada Research Chair in MSK Health Outcomes and Knowledge Translation Research Canada Ottawa, ON Canada; 8 Royal Society of Canada Ottawa Canada; 9 Lawson Research Institute St. Joseph's Healthcare London London, ON Canada; 10 Bone and Joint Institute Western University London Canada; 11 Department of Surgery Faculty of Health Sciences McMaster University Hamilton Canada; 12 Department of Family Medicine Faculty of Health Sciences McMaster University Hamilton Canada; 13 Psychiatry & Behavioural Neurosciences Department of Health Sciences McMaster University Hamilton Canada; 14 Michael G. DeGroote Cochrane Canada Centre at McMaster Faculty of Health Sciences McMaster University Hamilton, ON Canada; 15 Centre for Health Economics and Policy Analysis (CHEPA) Faculty of Health Sciences McMaster University Hamilton Canada; 16 ICES McMaster Institute for Clinical Evaluative Sciences Hamilton Canada

**Keywords:** mobility, self-management, task-oriented motor learning, preclinical mobility limitation, walking speed

## Abstract

**Background:**

Walking difficulties are a common and costly problem. However, disability associated with the decline in walking ability is not an inevitable consequence. With an aging population, it is increasingly important to establish strategies to help older adults preserve the capacity to live independently and function well in late life. Preclinical mobility limitation (PCML), which is characterized by subtle changes or limitations in mobility that precede disability, manifests as changes in how daily tasks such as walking are performed. Persons with PCML are at increased risk for the onset of disability and chronic disease. For that reason, PCML is a critical stage in the natural history of functional change when there is the opportunity for primary prevention interventions.

**Objective:**

To evaluate the effect of a 12-week online mobility self-management program (Stepping Up) on mobility outcomes, including walking speed, aerobic exercise capacity, dual-task cost, physical functioning (balance and strength), balance confidence, extent of community mobility, self-management of mobility, and quality of life in older adults with PCML.

**Methods:**

This randomized controlled trial (RCT) will recruit 249 older adults (aged 55-75 years) screened for PCML. Participants will be assigned to the Stepping Up program, a telephone-based coaching walking program or an online chair-based yoga program. Programs will be delivered over 12 weeks and participants will undergo virtual assessments with a blinded physiotherapist at baseline, 12, 24, and 36 weeks. An economic evaluation will be conducted alongside this RCT.

**Results:**

A total of 253 participants were enrolled in the trial. Data collection commenced in August 2021 and will be completed in October 2025. Data analysis will begin in November 2025 and results will be published in the Summer of 2026.

**Conclusions:**

To our knowledge, PCML has not been addressed by primary prevention interventions that incorporate both task-oriented motor learning exercise and mobility self-management sessions. Results will establish if the Stepping-Up program has the potential to serve as a model for sustainable, accessible, and cost-effective programming for individuals with early mobility limitations.

**Trial Registration:**

ClinicalTrials.gov NCT04368949; https://clinicaltrials.gov/study/NCT04368949

**International Registered Report Identifier (IRRID):**

DERR1-10.2196/72585

## Introduction

### Background

In 2024, 1 in 5 Canadians is older than 65 years and many of these individuals are experiencing some change in their walking ability [1]. Sufficient walking speed is a cardinal indicator of the mobility required to fulfill most tasks that humans undertake. Slowing of walking speed is associated with increasing age and has been associated with both survival [2,3] and independence [4,5]. Walking difficulties represent a common and costly problem [2]. However, disability associated with a decline in walking ability is not an inevitable consequence of aging [6]. With an aging population, it is increasingly important to establish strategies to help older adults preserve the capacity to live independently and function well later in life. The development of cost-effective interventions for primary prevention of mobility limitation is of paramount importance to the health care system. Health care costs in a US Medicare sample were US $2773 per year higher for persons who report difficulty walking half a mile and US $3919 higher for persons who were unable to walk this distance [7]. Further studies have estimated that over a one-year period, persons who developed mild walking difficulties had higher health care costs (US $1128 per person), which has been estimated at a societal cost of US $3.6 billion for the 22% of older persons who report incident walking difficulties annually [8,9]. There is currently no comparable Canadian cost data available.

Mobility, the ability to move within one’s environment, is essential to maintaining independence [6]. Importantly, changes in mobility are frequently the first indication of functional decline [10,11]. The onset of mobility decline is characterized by frequent transitions between states of mobility independence and mobility limitation and represents a highly dynamic process that is often indicative of health status [10,12,13]. Mänty (2007) [14] hypothesized that major mobility disability develops through stages, beginning with preclinical mobility limitation (PCML) which is characterized by subtle changes or limitations in mobility.

PCML manifests early in the process of declining mobility and is not typically identified or acted upon by clinicians. The prevalence of PCML in community dwelling samples ranges 20%-40% [15] and 31%-55% in adults over 75 years [16]. These mobility limitations manifest as changes in how daily tasks such as walking are performed (slower speed and lower endurance). Persons with PCML are at increased risk for the onset of disability and chronic disease [17,18]. Persistent deterioration in mobility is a predictor of mortality and has been reported even in the absence of changes in activities of daily living over a two-year period [19]. Further, older persons with mobility limitations, including reduced gait speed, are at risk for falls [4,20]. Thus, PCML is a critical stage in the natural history of functional change with aging when there is the opportunity for primary prevention interventions [21,22].

Interventions to improve walking (gait) are usually impairment-focused, aimed to address issues such as strength, flexibility, and endurance [23]. However, recent evidence suggests that interventions aimed to optimize mobility in older adults must also address task-oriented motor sequence learning to improve walking skills [23]. Motor skill-based exercise needs to address several components to optimize benefits to walking ability that include (1) an identified movement goal, (2) movement that provides feedback about muscle ability, posture, and movement awareness, (3) repetition and practice to correct errors, and (4) altering the environment and practice conditions to select an optimal motor plan [23].

Two randomized controlled trials (RCTs) examining the effect of task-oriented motor learning (TOML) exercise on mobility delivered to older persons (mean age 77.2, SD 5.5 years) showed greater improvement in energy cost of walking, gait quality, walking confidence, and physical function compared with persons who participated in impairment-focused exercise [24,25]. A recent large scale RCT (n=298) in senior residential apartments found a significant difference between an intervention focused on coordination and timing versus a seated strength and flexibility control. The between-group difference on the 6MWT was 17 m (*P*=.03) and between-group difference on gait speed measured using an instrumented walkway was 0.05 m/second (*P*=.02) [26]. Using a TOML approach facilitates the re-education of basic functional movement patterns to a wide variety of dynamic activities and targets more integrated aspects of balance and mobility.

Self-management (SM) has been promoted to increase healthy behaviors and manage symptoms for persons with chronic conditions [27]. Studies have examined the use of SM to deliver rehabilitation-based principles and manage musculoskeletal issues associated with chronic conditions [27-29], but SM has not been used to address changes in mobility.

According to our research, PCML has not been addressed by tailored primary prevention interventions that incorporate both TOML and SM. From 2014-2016, we undertook a pilot RCT (n=89) to compare an intervention that combined TOML exercise and mobility SM principles (Stepping Up) to an existing community-based fall prevention program. In the pilot, we established the feasibility and acceptability of the intervention as well as preliminary effects [30]. We now propose a fully powered RCT to demonstrate that the Stepping Up program will improve walking in persons with PCML.

### Objectives

Our primary objective is to determine if a 12-week mobility program called Stepping Up is more effective than a telephone-based coaching walking program or a chair-based yoga program at improving walking speed, assessed using the Four-Meter Gait Speed (4MGS) test. Secondary objectives are to compare the effectiveness of the Stepping Up program to the telephone-based coaching walking program and the chair-based yoga program for the following outcomes: (1) gait speed at 24 and 36 weeks and (2) aerobic exercise capacity, dual-task cost, physical functioning (balance and strength), balance confidence, extent of community mobility, SM of mobility, and quality of life at 12, 24, and 36 weeks.

We will compare the cost-effectiveness of the Stepping Up program to the telephone-based coaching walking program and the chair-based yoga program with respect to health care usage costs (including hospitalizations and physician visits) and program-related costs over 36 weeks.

Focus groups will be conducted to discover how participants in the Stepping Up program develop their mobility skills in comparison to those in the telephone-based coaching walking program and chair-based yoga program.

## Methods

### Trial Design

This is a pragmatic RCT [31,32], evaluating a complex intervention and reflecting the heterogeneity of individuals living in the community with PCMLs who might benefit from this intervention. The SPIRIT (Standard Protocol Items: Recommendations for Interventional Trials) checklist is available in Multimedia Appendix 1. Both outcome assessors and data analysts will be blind to group allocation. Participants will be evaluated at 4 time points: baseline (0 weeks), at the end of the intervention (12 weeks), and at follow-up (24 and 36 weeks).

### Study Setting

The Stepping Up and chair-based yoga interventions will be delivered in synchronous online classes, and the telephone-based coaching walking intervention will be delivered by phone. This trial was initially planned for in-person delivery but switched to online delivery due to COVID-19 restrictions.

### Eligibility Criteria

Potential participants will initially be screened by phone and subsequently via videoconferencing by the research coordinator (RC) who is a physiotherapist. Inclusion criteria include (1) community-dwelling, (2) age ≥55-75 years, (3) PCML as assessed using a questionnaire that requires respondents to self-report difficulties with their mobility (walking 2 km). Respondents will be considered in a stage of PCML if they report no task difficulty but report modification of task performance (ie, modify frequency, method, or time to complete the task) [14], (4) understanding of spoken and written English, (5) own a laptop, computer, or tablet and have an email address and the internet capabilities of running the videoconferencing platform, Zoom (Zoom Video Communications). We will exclude persons (1) with a score of <11 on the Montreal Cognitive Assessment 5-minute telephone screen [33], (2) with a major illness that would prevent participation, (3) who are not residents of Ontario, and (4) receiving active physiotherapy treatment.

Following screening by telephone, each potential participant will be emailed a copy of the information letter and consent form. During an introductory Zoom session with each participant, the RC will review the information letter, answer all questions, and obtain verbal consent. The RC will keep a verbal consent log and participants will be mailed a signed copy of the consent form, indicating that verbal consent was provided. Ongoing consent will be implied unless participants withdraw from the study. Participants will be asked not to participate in other studies delivering an exercise intervention during enrollment in the trial.

### Interventions

The Stepping Up program is a 12-week, multicomponent intervention that includes tailored TOML exercise and a mobility SM program developed for the pilot study. Participants will attend two 1-hour synchronous online sessions (1-hour exercise and 1-hour SM) per week using Zoom. The exercise component includes a variety of stepping sequences that are intended to enhance timing and coordination and improve automatic control of walking. Timing and coordination problems impact walking efficiency and contribute to age-related decline in physical function for older adults. [24] The exercise program begins with simple stepping patterns and progresses over 12 weeks. Each participant has a card which indicates the tailoring to address individual difficulties and mastery of each stepping pattern, as well as the progression to increase the complexity. Progression involves introducing more complex stepping tasks (ie, diagonal patterns that require stepping across midline), as well as increasing speed and varying the base of support. Once a stepping pattern is mastered, a dual task is introduced (ie, carrying an object in one or both hands and performing a cognitive task). The online class will be led by a fitness instructor and monitored by a physiotherapist who will provide feedback and individually tailor the exercises for each participant. The mobility SM component, facilitated by a physiotherapist, will aim to enhance participants’ understanding of (1) changes in mobility with aging [17,34], (2) active SM of mobility including self-monitoring [27,28,34], (3) preclinical disability [17,18,35], (4) the importance of walking speed [2,23], (5) fall prevention [36], (6) exercise and TOML to improve mobility [25,37], (7) strategies to optimize sleep [38-40], (8) managing pain and physical discomfort [41,42], (9) conserving energy and minimizing fatigue [43], (10) better breathing [44], (11) posture to promote mobility [45,46], and (12) environmental barriers to walking and mobility [47,48]. The SM sessions were developed based on research evidence, discussion in an online forum with physiotherapists and researchers in mobility and aging, and in consultation with older adults. The SM component aims to increase self-efficacy through collaborative goal setting and action planning. A study website will allow participants to view short videos of the walking patterns being performed and review the SM sessions.

We will incorporate two control groups to establish the true effect of the Stepping Up intervention. The telephone-based coaching walking program will act as a comparative effectiveness control group. A review of interventions to increase walking behavior found that telephone interventions as brief prompts appeared to be as effective as in-depth counseling supporting walking prescriptions of 5-7 days per week versus 3-5 days per week [49]. In these studies, telephone interventions were often added as an adjunct to the primary intervention and mobility was not always measured as an outcome. Telephone counseling, compared with no intervention, has been shown to increase physical activity in low-activity older persons recruited from primary care [50,51]. However, it is unclear whether telephone-delivered interventions are cost-effective compared with face-to-face interventions for improving walking in persons with PCML [52].

The telephone-based coaching walking program will be delivered by a physiotherapist. The frequency, duration, and intensity of walking will be determined based on the individual’s current level of physical activity and an end target level of physical activity (minimum of 150 minutes of moderate-intensity aerobic exercise per week) [53]. The physiotherapist will engage participants in goal setting and action planning, which will include discussion of coping, planning, identifying support systems, and evaluating self-efficacy. The initial telephone contact will last 20-30 minutes, and subsequent weekly calls will last approximately 10 minutes. Therapists will help participants evaluate their action plans, assist with problem-solving, discuss program progression, and help develop new action plans. This group will also receive written material about the benefits of walking and the importance of walking speed.

The chair-based yoga program will act as an attention control group. Chair-based yoga programs are typically reserved for older adults who are unable to participate in regular exercise or standing yoga programs. An RCT evaluating an 8-week chair-yoga program for older adults with lower extremity osteoarthritis who could not participate in standing exercise found that participants experienced a reduction in pain and an increase in gait speed compared with those in a health education program [54]. These improvements were not sustained postintervention. Chair-based yoga has not been evaluated for effectiveness in improving mobility outcomes for higher-functioning older adults with PCML.

Participants in the chair-based yoga program will undertake chair-based yoga sessions that include breathing control, seated poses, standing poses, and relaxation exercises, led by a fitness instructor. Participants will join a synchronous online class for 1 hour, twice per week for 12 weeks.

### Outcomes

Demographic information (birth date, sex, gender, marital status, living accommodation, employment status, education, ethnic origin, and household income) and comorbid conditions (identified using the Self-Administered Comorbidity Questionnaire) [55] will be collected at baseline. Primary and secondary outcomes will be assessed virtually at baseline, 12 weeks, 24 weeks, and 36 weeks. Health care usage data will be collected during follow-up assessments only.

### Primary Outcome: Walking Speed

The primary outcome measure is walking speed. The 4MGS test [56] is a performance-based measure of walking speed. The 4MGS test will be performed from a standing start at both a self-selected (usual) and fastest walking speed [57]. Although usual walking speed is more commonly reported in the literature [58], there has been some evidence in which maximum walking speed outperforms usual walking speed in identifying preserved exercise capacity [59]. Furthermore, maximum walking speed has been found to be a better predictor of future dependence in activities of daily living (eg, bathing, dressing, walking, and eating) in individuals aged 65-74 years and usual walking speed to be a better predictor of future dependence in activities of daily livings in individuals older than 75 years of age [60]. The 4MGS test has excellent test-retest reliability (intraclass correlation coefficient [ICC] 0.96) in healthy older adults [56]. 4MGS is responsive to clinically meaningful changes with 0.05 m/second denoting a small change and 0.1 m/second indicating a substantial change [61].

### Secondary Outcomes

#### Dual Task Cost

Dual task cost will be measured using the dual-task Timed Up and Go (TUG) [62]. This test requires individuals to stand up from a chair, walk 3 m at usual speed, cross a line marked on the floor, turn around, walk back, and sit down while counting backward by threes from a randomly selected number between 20 and 100 [62]. Dual task cost is calculated as the difference in time (in seconds) between the TUG and the Dual-Task TUG. A systematic review identified that changes in gait speed over varying distances (3, 5, and 25 m) under dual-task rather than single-task conditions were associated with fall risk in community-dwelling older adults [63-65]. It has strong interrater reliability (ICC 0.94) and concurrent validity with the Berg Balance Scale (*r*=–0.66) [64]. There are no reports on the minimal detectable change for the Dual-Task TUG, but the minimal detectable change for the TUG is between 3.5 and 4.1 seconds [66,67].

#### Exercise Capacity

The 2-minute step test is a test of exercise capacity that can be used as an alternative to a timed long-distance walking test when assessment space is limited [68]. Individuals are required to march in place as quickly as possible for 2 minutes while lifting their knees to a height midway between their patella and iliac crest when standing [69]. Both knees must be raised to the correct height to be counted. The score is the total number of times the right knee reaches the minimum height target. Rikli and Jones [69] found the 2-minute step test demonstrated good test-retest reliability (ICC 0.90) and convergent validity relative to 1-mile walk time (*r*=0.73) in older adults.

#### Balance

The Unipedal Stance Test is used to assess static balance [70]. Individuals are asked to stand barefoot on the limb of their choice with arms at their sides until they (1) use their arms (ie, move their arms away from their sides), (2) use their raised foot (ie, move it toward or away from the standing limb or touch the floor), (3) move the weight-bearing foot to maintain their balance (ie, rotate foot on the ground), or (4) maintain the position for a maximum of 45 seconds. The procedure is repeated 3 times, and the best and average time (s) of the 3 trials is recorded. The UPST has excellent inter-rater reliability for both the best of 3 trials ICC 0.994 (95% CI 0.98-0.996) and the mean of 3 trials ICC 0.951 (95% CI 0.926-0.969) in older adults [70].

#### Lower Extremity Strength

The 30-Second Chair Stand Test is used to assess lower body strength and power and is measured by the number of chair stand repetitions in a 30-second period. It has excellent test-retest reliability (*r*=0.89, 95% CI 0.79-0.93), interrater reliability (*r*=0.95, 95% CI 0.84-0.97), and concurrent validity with leg press performance (*r*=0.77, 95% CI 0.64-0.85) [69].

#### Self-Perceived Mobility

The Mobility Assessment Tool-Short Form (MAT-sf) is a video-animated tool for assessing mobility. It consists of 12 animated video clips that assess an individuals’ perceived level of proficiency in performing each mobility task [71]. Participants are asked to estimate their ability to perform each task by responding yes or no, or by indicating the number of minutes or number of times they can complete the task. The possible range of scores is from 30 to 80 with higher scores indicating better mobility. The MAT-sf demonstrates excellent test-retest reliability (ICC 0.93, *P*<.001) and evidence of known groups validity (older adults who were able to complete the 400-m walk test have higher MAT-sf scores than those who failed the test, *P*<.001) [71].

#### Change in Mobility

The Global Mobility Change Rating consists of a single question: “Since your last visit, has there been any change in your mobility?” The response will be made on a 11-point self-reported Likert scale: –5=very much worse, 0=unchanged, and 5=very much better [72]. These scales have excellent test-retest reliability (ICC 0.90) and a minimally clinically important difference (MCID) of 2 points on an 11-point scale [72].

#### Preclinical Mobility Limitation

The PCML Scale allows for categorization into 4 groups of self-reported mobility limitation based on participants’ ability to walk 2 km: (1) no mobility limitation, (2) PCML, (3) minor manifest mobility limitation, and (4) major manifest limitation [14]. The risk ratio for the development of major manifest mobility limitation in walking 2 km for persons with PCML at baseline compared with persons with no mobility limitation at baseline was 5.8 (95% CI 2.6-12.9) [14]. The PCML Scale will be administered at 12, 24, and 36 weeks and changes in the level of mobility limitation will be evaluated between time periods using chi-squared tests.

#### Physical Activity Level

The International Physical Activity Questionnaire (IPAQ) is a 27-item self-administered questionnaire that is used for monitoring physical activity and inactivity [73]. It collects information about the frequency and duration of sitting, walking, moderate and vigorous activity during a 7-day period. It has excellent test-retest reliability for overall score (ICC 0.81) and physical activity (ICC 0.84). It has excellent concurrent validity (correlations of time spent in vigorous physical activity compared with accelerometer monitoring [ρ=0.71]) and adequate concurrent validity (correlations of time spent in physical activity at home and during leisure time compared with logbook monitoring [ρ=0.47, ρ=0.58, respectively]) [74].

#### Self-Reported Balance Confidence

The Activities-specific Balance Confidence Scale is a self-report measure of balance confidence [75]. Individuals are asked to rate their confidence in completing 16 common tasks without losing balance, on a scale from 0% (no confidence) to 100% (complete confidence). The Activities-specific Balance Confidence Scale has demonstrated excellent test-retest reliability (*r*=0.92) and has been shown to have better scale responsiveness than the Falls Efficacy Scale when used with community-dwelling older people aged 65-95 years [75].

#### Pattern of Mobility

The Life-Space Assessment measures a person’s usual pattern of mobility during a 1-month period, documenting mobility based on how far and how often a person travels and any assistance required [76]. The Life-Space Assessment has excellent test-retest reliability (ICC 0.96) [77] and has been used in longitudinal studies of aging [76] to assess mobility frequency and distance. It will be used to determine baseline levels of self-reported mobility and to track changes that occur with the interventions.

#### Knowledge, Skill, and Confidence for SM

The Patient Activation Measure is a 13-item measure of the patient’s level of knowledge, skill, and confidence for SM [78]. Raw scores (13-52) are converted into activation scores (0-100) with higher scores indicating greater patient activation. The Patient Activation Measure has a MCID of 0.5 points [79] with high test-retest reliability (ICC 0.85) and evidence of validity against a classification of individuals’ level of activation (κ=0.8-0.9) [80].

#### Quality of Life

The EQ-5D-5L is a generic utility-based health related quality of life questionnaire [81] with an MCID of 0.056 [82]. It consists of 5 questions covering the dimensions of mobility, self-care, usual activities, pain and discomfort, and anxiety and depression with 5 response options indicating no, mild, moderate, severe, and extreme problems for each dimension [83]. The EQ-5D-5L can define a total of 3125 health states which can be converted to a utility index anchored at 0 for a state being equal to dead, and 1 for full health using the Canadian Scoring function [84]. The utility index combined with life expectancy allows for the estimation of quality-adjusted life years (QALYs), a recommended generic outcome measure in health economic evaluation in Canada and other countries [85-87].

#### Self-Efficacy for Physical Activity

Participants will be asked to rate how confident they are that they could participate in moderate intensity physical activity for 150 minutes per week using a single question on a scale of 1 (not at all confident) to 10 (completely confident) [88].

#### Health Care Usage

Health care usage data to assess service use and cost effectiveness of the trial will be collected using administrative data accessible at the Institute for Clinical Evaluative Sciences (ICES) at McMaster University. Specifically, usage data will be obtained through the following databases: Ontario Health Insurance Plan, National Ambulatory Care Reporting System, and the Discharge Abstract Database, which are all housed at ICES McMaster. We will use standard person-level costing approaches established by ICES [89] to capture the formal component of direct health care costs. Health care usage data will also be collected using a case report form (CRF) to complement the ICES administrative data. If a variable is not available in an administrative database, the CRF will capture the variable and be included in the economic analysis using standard micro-level costing approaches. Health care usage variables that will be collected using the administrative databases and CRF are direct costs, including health care facility visits, hospitalizations, family doctor visits, specialist visits, tests and procedures, other health care services, and falls. The project leads will work with ICES scientists, a health economist, and an ICES Research Analyst. The administrative databases will be accessed once all of the clinical data has been collected.

#### Focus Groups

Our pilot study demonstrated perceived transformative aspects of the intervention that require further exploration and explication. Focus groups, following an interpretive descriptive approach, will be conducted [90] to identify possible aspects that enable participants to improve their mobility and become more successful self-managers and explore how these may differ in the Stepping Up intervention compared with the telephone-based coaching walking program and chair-based yoga program. Gender considerations will be explored to describe how changes in mobility self-efficacy and SM are related to gendered environments and gendered role expectations. Virtual focus groups (4-6 per group) will be conducted by a qualitative researcher. Participants will be invited to take part in the focus groups at the end of the 12-week intervention period. There will be a total of 4 focus groups for each of the 3 interventions for a total of 12 focus groups over the course of the study. Interviews will be audio-recorded, transcribed, and deidentified. Refer to Multimedia Appendix 2 for the interview guide.

#### Sample Size

The sample size calculation is based on the primary outcome measure, the 4MGS test. Small clinically meaningful change is 0.05 m/second on the 4MGS test in older adults [2]. We expect to observe minimal to no change in the chair-based yoga group (0 m/s), small but meaningful change in the telephone-based coaching walking group (0.05 m/s), and a large change in the Stepping Up group (0.12 m/s) [61]. Assuming a correlation between baseline and 12-week scores of 0.5, a mean baseline gait speed of 1.14 m/second, and a common SD of 0.22, a sample size of 66 per group (N=198) will have 80% power at the 0.05 significance level. To account for dropouts (20%), the planned total sample size is 249 (or 83 per group). Sample size calculations were performed using SAS (version 9.4; SAS Institute).

### Recruitment

**Figure 1:**
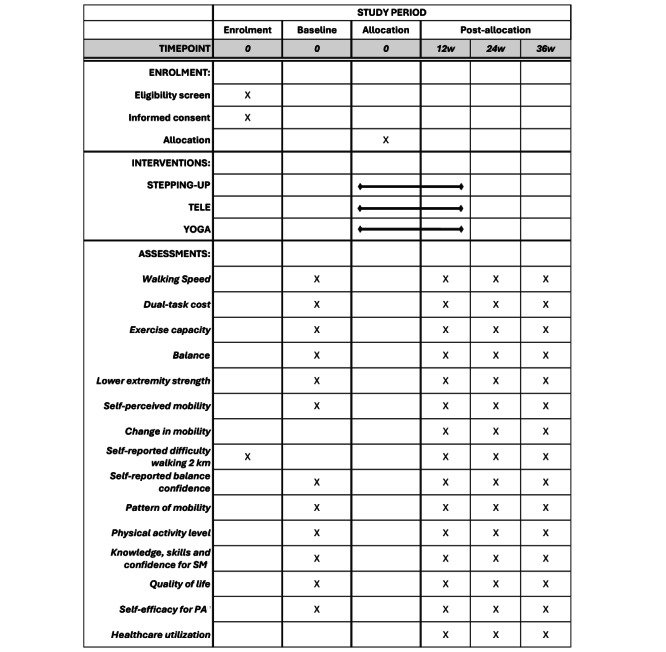
SPIRIT (Standard Protocol Items: Recommendations for Interventional Trials) schedule of enrollment, interventions, and assessments. SM: self-management; PA: physical activity.

Participant recruitment will occur from August 2021 to January 2025, and follow-up assessments will be completed by October 2025. Refer to Figure 1 for the SPIRIT schedule of enrolment, interventions, and assessments. Participants will be recruited through advertisements in local newspapers and social media sites (ie, Facebook and Instagram, Meta). We will send out promotional material about the study in e-Newsletters of agencies that cater to older adults (ie, senior research centers, university retiree groups, and local Councils on Aging), and advertise on the websites of local libraries and Active Aging Canada. Posters and postcards will be displayed in local Young Men’s Christian Associations (YMCAs), helping to spread the word and raise awareness for the study. The ads will be created using design elements that appeal to older adults and will include information about how to contact the research team. We will recruit over a period of 42 months (recruitment rate of 6-8 participants per month), and we will deliver the interventions to groups of 5-7 participants in 12-week sessions.

### Randomization

The unit of randomization will be the participant, and the randomization process will use computer generation of group assignment using the REDCap (Research Electronic Data Capture; Vanderbilt University) randomization module. REDCap is a secure web application designed to support data capture for research studies [91]. Participants will be stratified by age (55-64 years or 65-75 years). An allocation table will be created and uploaded to the REDCap randomization module by a statistician not involved in the RCT. Using a 1:1:1 allocation ratio, we will block the randomization with variable block sizes of 3, 6, and 9. The RC will not have access to the allocation table. Once participant consent is obtained and the baseline assessment is complete, the RC will access the randomization module in REDCap to enter the participant strata value (age category) and receive the group allocation.

### Data Collection and Management

During an introductory Zoom session with each participant, the RC will discuss space requirements and setup for the assessments and review emergency procedures should an adverse event occur. Written instructions will be sent by email before each assessment. The instructions will describe the setup required for the assessment and a list of equipment needed (measuring tape, chair, and masking tape to mark distances). If the participant is unable to secure these items independently, they will be mailed to them before their baseline assessment.

The same physiotherapist blinded to group assignment will perform baseline and follow-up assessments for a given participant. All trial assessments will be conducted virtually using the Zoom platform. During the assessments, assessors will use screen-sharing to administer patient-reported questionnaires and enter participant responses directly into REDCap. For the performance measures, assessors will use screen-sharing to play a video clip to demonstrate how the participant is expected to complete each of the physical tests that will be administered. Participants will then complete each test with the assessor observing by video, and scores will be entered into REDCap.

We have estimated the rate of loss to follow-up in our sample size calculation as 20%. We will attempt to minimize loss to follow-up in the trial by using a 2-stage screening process and by having the RC follow-up with participants after 2 consecutive unreported absences. Participants who drop out of their intervention will be given the opportunity to complete follow-up assessments. Those participants who complete follow-up will also be offered a personalized report that provides them with a summary of their results.

Intervention-specific data will be entered into REDCap by trained study staff or by survey. The physiotherapist delivering the telephone-based coaching walking intervention will enter data pertaining to the participant’s goals and action plans during the weekly telephone sessions. Participants in the Stepping Up intervention will receive weekly REDCap surveys and will record their goals, action plans, and practice minutes. The RC will perform regular checks to ensure completeness and will follow-up on any missing data.

Personal information will be collected directly from each participant by the RC during the introductory Zoom session. Personal information (name, address, phone number, and phone number of an emergency contact) will be shared with the assessors using a private Google Calendar and with the study staff delivering the interventions using encrypted email. Participant data will be deidentified using a study ID number. A password protected electronic file linking participant identifiers to their study ID number will be stored on a secure server. Only the Primary Investigators and the RC will have access to this file. Additionally, only the primary investigators and authorized users will have access to the project in REDCap. User rights will be configured to ensure that users only have access to the data essential to their role.

### Analysis

Baseline characteristics will be analyzed using descriptive statistics reported as mean (SD) or median (first quartile {Q1}, third quartile {Q3}) for continuous variables, depending on the distribution and count (%) for categorical variables. All analyses of primary and secondary outcomes will follow the intention-to-treat principle. The results will be reported as relative risk or odds ratio for binary outcomes or mean difference for continuous outcomes, with corresponding 95% CI and associated *P* values. All *P* values will be reported to 3 decimal places with those less than .001 reported as *P*<.001. All analyses will be performed using SAS 9.4. We will use multiple imputation to handle missing data [92].

To assess the effect of the intervention on the primary outcome (4MGS), we will conduct an analysis of covariance adjusting for age as a stratification variable and baseline value of 4MGS as a covariate. Secondary outcomes will also be analyzed using analysis of covariance, again using age as a stratification variable and baseline value for each outcome as a covariate. No interim analysis is planned.

We will conduct exploratory subgroup analysis for both the primary and secondary outcomes by including an interaction term between the subgroup variable (sex and age) and the treatment group variable. The multiple measures subgroup analyses will not be adjusted for multiple testing since they are exploratory.

Our hypothesis is that there will be reduced hospitalizations and physician visits for the intervention group compared with the control groups. Cost data is usually treated as nonparametric. Therefore, a Poisson or negative binomial distribution will be used to model the number of hospitalizations and physician visits by counts to determine the difference between the groups on relative intervention cost.

Analysis of the qualitative interview transcripts will follow an interpretive descriptive approach [90], an approach used in applied health disciplines to inform practice. A member of the research team will lead the analysis involving: (1) multiple line by line readings of the data to ensure a deep understanding of the data; (2) an inductive and deductive coding process based on the research questions and the emerging analysis; (3) the use of a data matrix to facilitate the manipulation of coded data and exploration of patterns, variations, and discrepancies, as well as comparisons within and among the participant groups in the data; and (4) finally, asking questions of the data to understand aspects of intervention and to answer the research questions. Rigor will be enhanced by the maintenance of a detailed record of all steps [93], inclusion of multiple perspectives in the analysis [94], guidance of a methodological framework [90], and use of the NVivo qualitative data analysis software (Lumivero) [95].

The cost-effectiveness analysis will compare the Stepping Up program with the telephone-based coaching walking program and the chair-based yoga program from the public health care payer’s perspective. The main effectiveness measure for the cost-effectiveness analysis will be the incremental cost per QALY gained over 36 weeks. We consider 36 weeks’ sufficient time to capture the potential cost-effectiveness of Stepping Up. As this cost-effectiveness analysis is conducted alongside the trial, nonparametric bootstrap will be used to calculate the 95% CI around the incremental cost per QALY ratio. The decision uncertainty will be presented using cost-effectiveness acceptability curves, which show the probability of the intervention being cost-effective compared with the telephone-based coaching walking group and the chair-based yoga group.

### Oversight and Monitoring

A steering committee will be established comprised of all central study team members, older adults from the community, and partners from the YMCA. The steering committee will review study progress and monitor recruitment, data quality and completeness, and general study conduct. The steering committee will monitor the rate of adverse events and will be responsible for the dissemination of results and will meet every 6 months. Given that this trial presents minimal risks, we will not have a data monitoring committee.

We do not anticipate any safety risks to the participants. However, it is possible that they might experience muscle stiffness, soreness, falls, or injuries. Since the interventions will be delivered remotely, each participant will provide the name and telephone number of a friend or family member that the research staff can contact should the participant be alone at home and an emergency arises. If the adverse event is deemed to be the result of the intervention, an Adverse Event Form will be completed by a member of the trained research staff and appropriate medical attention will be sought as necessary.

### Ethical Considerations

This study has received final approval from the Hamilton Integrated Research Ethics Board (HiREB Project #10663). Participants will provide informed verbal consent to participate. Ongoing consent will be implied by continued involvement in the trial. Participants will be informed that they may withdraw at any time with the option of removing their data from the study by notifying study staff.

## Results

**Figure 2:**
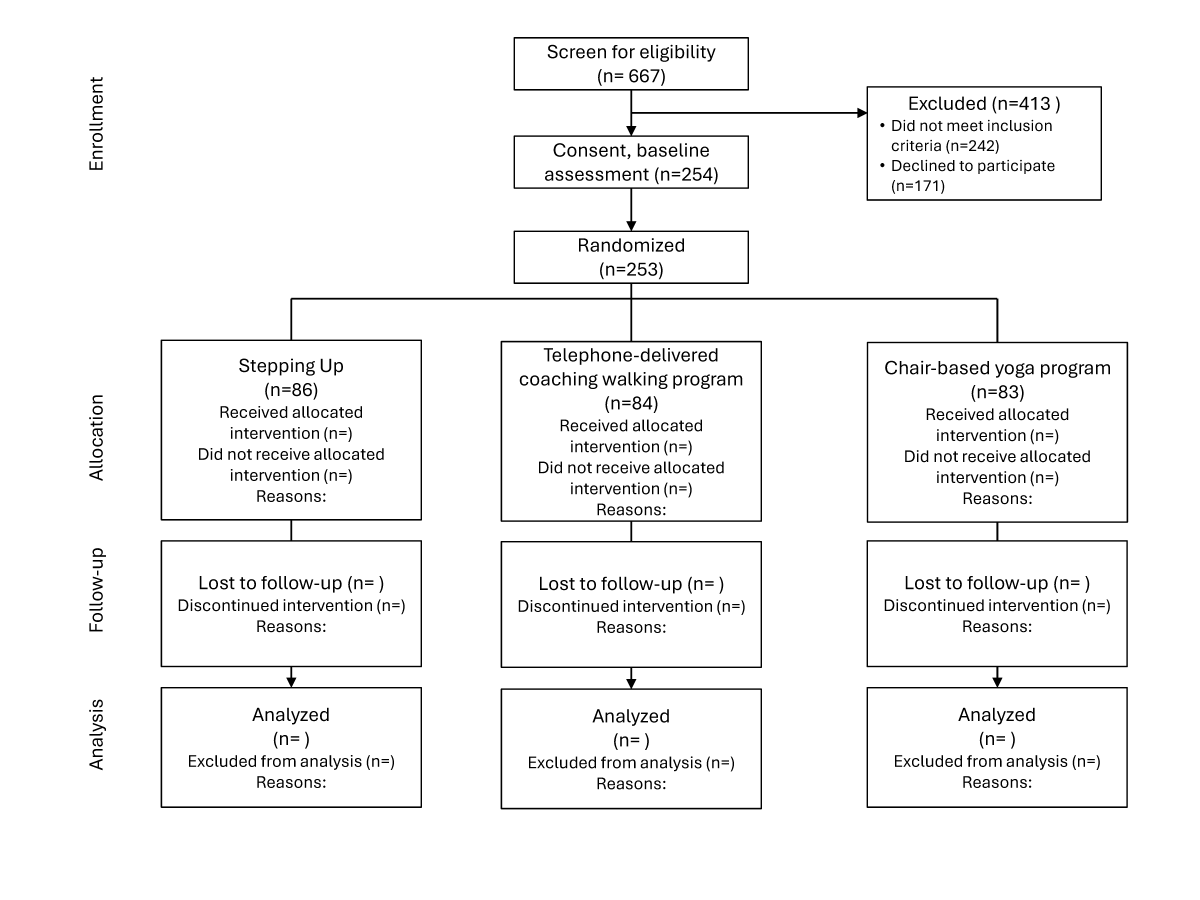
CONSORT (Consolidated Standards of Reporting Trials) flow diagram.

Recruitment for the trial concluded in January 2025 with a total of 253 participants enrolled. Data collection commenced in August 2021 and will be completed in October 2025. Data analysis will begin in November 2025 and results will be published in Summer 2026. Refer to Figure 2 for the CONSORT flow diagram. Data supporting this study will be openly available from the Open Science Framework repository [96].

## Discussion

### Principal Findings

Approaching functional decline from a primary prevention perspective by targeting individuals with PCML is important. Online community-based mobility programs have the potential to reach large groups of older adults to improve quality of life and to maintain or enhance physical function [97]. Such programs may also translate to lower health care system costs [98]. There have been several other rehabilitation programs successfully delivered online with positive results, including a multicomponent rehabilitation program to frail older adults during the pandemic [99], a cardiac rehabilitation program [100], and stroke [101] and cancer [102] rehabilitation programs. A recent survey showed that 71% of the 184 pulmonary rehabilitation programs surveyed were delivered remotely [103].

The results of this trial will inform future research and clinical practice. We will integrate knowledge translation activities such as the synthesis, exchange, dissemination, and application of knowledge to provide more effective health care services through complex interactions between researchers and knowledge users [104]. We will collaborate with the YMCA, with whom we have a long-term relationship, and they will be actively involved in the ongoing delivery of the study. LiveWell is a strategic partnership between McMaster University, the YMCA, and Hamilton Health Sciences whose mission is to promote health through community access to innovative, evidence-based wellness programs. This partnership will allow us to share our results with other YMCAs whose core business involves the promotion of activity, mobility, and healthy communities. Furthermore, the program will be available in an open-source format. Knowledge users, with the capacity to promote adoption of the results, will be closely involved in the project and include a member of the McMaster Optimal Aging Portal Expert Advisory Committee (JR) and the Senior Regional Manager of the YMCA of Hamilton, Burlington and Brantford, Health and Fitness Division. The Nominated Primary Investigator (JR) has a strong network within primary care provincially and nationally, and as a member of the Primary Care Advisory Committee to the Ontario Physiotherapy Association, she will lead dissemination within this sector. We will also share results through the McMaster Optimal Aging Portal and through publications that target older adults who may be experiencing preclinical mobility problems, as well as through high impact internationally peer-reviewed journals. We have 3 older adult community members who are collaborating on this project and who have been involved in the development of this intervention. These individuals will continue to consult on the execution of the study and will be involved in the development of knowledge translation materials and the dissemination of results.

### Limitations

There are some limitations that might be encountered during this trial. The delivery of the intervention virtually rather than in person may result in problems detecting when a participant is having difficulty and requires correction. The ability to provide individual tailoring or to determine if a participant has achieved mastery of an exercise will be challenging in an online group format since participants will be progressing at different rates and 1-1 interactions will disrupt the flow of an online class. To mitigate these issues, we will have the physiotherapist observing and providing feedback and tailoring recommendations to the participants at the end of the class.

### Conclusions

This trial will evaluate the impact of the Stepping Up program on walking speed, as well as on aerobic exercise capacity, dual-task cost, physical functioning (balance and strength), balance confidence, extent of community mobility, SM of mobility, and quality of life. We will also examine the cost-effectiveness of the STEPPING UP program. Results will establish if this evidence-based initiative, which has modest resource demands, has the potential to serve as a model for sustainable, accessible, and cost-effective programming for individuals with early mobility limitations.

## Appendix

Multimedia Appendix 1 SPIRIT (Standard Protocol Items: Recommendations for Interventional Trials) checklist.

Multimedia Appendix 2 Interview guide.

## Abbreviations

4MGS: Four-Meter Gait Speed
CRF: case report form
ICC: intraclass correlation coefficient
ICES: Institute for Clinical Evaluative Sciences
IPAQ: International Physical Activity Questionnaire
MAT-sf: Mobility Assessment Tool-Short Form
MCID: minimally clinically important difference
PCML: Preclinical mobility limitation
QALY: quality-adjusted life year
RC: research coordinator
RCT: randomized controlled trial
REDCap: Research Electronic Data Capture
SM: self-management
SPIRIT: Standard Protocol Items: Recommendations for Interventional Trials
TOML: task-oriented motor learning
TUG: Timed Up and Go
YMCA: Young Men’s Christian Association

## Data Availability

The datasets generated and analyzed during this study are available in the Open Science Framework repository.
