# Supplementary material for: Effect of an Online Mobility Self-Management Program on Walking Speed in Older Adults With Preclinical Mobility Limitation: Protocol for a Randomized Controlled Trial
Source: JMIR Res Protoc. 2025 May 21;14:e72585. doi: 10.2196/72585 (PMC12138319; doi:10.2196/72585)
Supplement: Multimedia Appendix 2 [file resprot_v14i1e72585_app2.docx]

**Intervention Group: STEPPING-UP Program**

1. You have completed your participation in the Stepping-Up program. If a friend asked you to describe this program, what would you say?
2. Tell me about the group environment for self-management and the exercise sessions. What was that like?
   1. Did you find the learning in a group format helpful? How so?
   2. Did you find doing the exercises in a group helpful? How so?
   3. How do you think the group format compares to experiences you’ve had doing exercise on your own?
   4. Do you think that you get more out of the group? Can you tell me more?
3. This program uses stepping exercises to improve mobility. How did you find this approach in comparison to other types of exercise that you’ve used in the past?
4. How is what you have learned in this program different from the way you usually manage your health and mobility/walking?
   1. Do you think that what you learned in this program has made you look at your health and mobility/walking differently?
   2. Do you think that it has made you consider how you get around in a different way?
5. How do you feel after 12 weeks of exercise?
   1. Do you think that you move around differently now? Prompt: If they say yes, ask more details e.g. indoors? Outdoors? Moving around their home? Do you feel that you have more energy? More strength or endurance?
   2. What do you attribute this change to? Prompt: Why do you think there was no change?
6. How did you use the information you learned in the self-management sessions?
   1. How did you incorporate any skills you learned into everyday life?
7. There were different resources available to you during the 12-week program. You had access to a website and we provided a log book. Did these resources and methods of support help you?
   1. Prompt for each, the website; the participant log.
   2. For each one, if so, ask how did they help?
8. What are the strengths of the program? What were your favourite aspects of the program?
9. What are the weaknesses of the program? Can you describe what aspects of the program that you did not like as much or that you would prefer not to participate in?
   1. How might the program be altered to better meet your needs?
10. Do you think that you’ve made any personal changes as a result of participating in this program?
    1. How have you changed?
11. Would you recommend this program to a friend?
    1. If so, to what kind of friend would you recommend this program? (e.g. someone with health challenges? Someone wanting to be more aware of how to exercise? Etc). Why?

**Comparative Effectiveness Control Group: TELE Program**

1. You have been receiving advice from a physiotherapist about a walking program for a number of weeks now. If a friend asked you to describe this intervention, what would you say?
2. Tell me about receiving this coaching over the phone. What was that like?
   1. Did you find that talking over the phone helpful or a good way of doing this?
   2. Did talking on the phone present any barrier in comparison to being in person?
3. How does this program differ from, or add to, the way you usually manage your health and mobility?
   1. Do you think that what you learned in this program has made you look at your health differently?
   2. Do you think that it has made you consider how you get around in a different way?
4. What are the strengths of this kind of program? What were your favourite aspects of the program?
5. How do you feel after 12 weeks of exercise?
   1. Do you think that you move around differently now? Prompt: If they say yes, ask more details e.g. indoors? Outdoors? Moving around their home? Do you feel that you have more energy? More strength or endurance?
   2. What do you attribute this change to? Prompt: Why do you think there was no change?
6. What are the weaknesses of this kind of program? What aspects of this program did you not like as much?
   1. How might this program be altered to better meet your needs?
7. Do you think that you’ve made any personal changes as a result of participating in the TELE program?
   1. How have you changed?
8. Would you recommend the TELE program to a friend?
   1. If so, to what kind of friend would you recommend this program? Why? (e.g. someone with health challenges? Someone wanting to be more aware of how to exercise? etc). Why?

**Placebo Control Group: YOGA Program**

1. You have completed the Chair-Based Yoga program. If a friend asked you to describe this program, what would you say?
2. Tell me about the group environment for the yoga sessions. What was that like?
   1. Did you find doing the yoga in a group helpful? How so?
   2. How do you think the group format compares to experiences you’ve had doing yoga or exercise on your own?
   3. Do you think that you get more out of the group? Can you tell me more?
3. How does this program differ from, or add to, the way you usually manage your health and mobility?
   1. Do you think that what you learned in this program has made you look at your health differently?
   2. Do you think that it has made you consider how you get around in a different way?
4. How do you feel after 12 weeks of exercise?
   1. Do you think that you move around differently now? Prompt: If they say yes, ask more details e.g. indoors? Outdoors? Moving around their home? Do you feel that you have more energy? More strength or endurance?
   2. What do you attribute this change to? Prompt: Why do you think there was no change?
5. What are the strengths of this kind of program? What were your favourite aspects of the program?
6. What are the weaknesses of this kind of program? What aspects of this program did you not like as much?
   1. How might this program be altered to better meet your needs?
7. Do you think that you’ve made any personal changes as a result of participating in the YOGA program?
   1. How have you changed?
8. Would you recommend the YOGA program to a friend?
   1. If so, to what kind of friend would you recommend this program? Why? (e.g. someone with health challenges? Someone wanting to be more aware of how to exercise? etc). Why?
